# Pain relief in plantar fasciitis within 6–8 weeks using (ortho)manual therapy of the foot joints: a prospective cohort study

**DOI:** 10.7717/peerj.21280

**Published:** 2026-05-25

**Authors:** Johanna Gerhardina van der Linde, René Somers, Maarten Alexander van Egmond

**Affiliations:** 1 VoetPortaal, Amsterdam, Netherlands; 2 School of Physiotherapy, Faculty of Health, Sport and Physical Activity, Amsterdam University of Applied Sciences, Amsterdam, Netherlands

**Keywords:** Mechanical overload, Plantar fasciitis, Manual therapy, Foot biomechanics, Patient care optimization, Correction soles

## Abstract

**Objectives:**

What changes over time are measured on pain, activities, quality of life and thickness of the plantar fascia when (ortho)manual therapy of the foot joints is applied to patients with plantar fasciitis (PF).

**Methods:**

In this observational single-arm cohort study, 50 patients with PF (31 females and 19 males, aged between 27 and 73 years) were recruited over a 22-month period. Of the 50 patients included, 32 completed the 26-week follow up. Eight patients were excluded after T2 because they needed additional spine treatments. 10 dropped out during the remainder of the study. The intervention protocol involved five visits in the first 6–8 weeks for assessments and treatments and one visit after 26 weeks for evaluation. The (ortho)manual therapy consisted of manual manipulations of the foot joints often preceded by the techniques with hammer and thrift. The (ortho)manual therapy was combined with a corrective insole. Primary outcomes were pain intensity numeric pain rating scale (NPRS), and plantar fascia thickness, assessed via ultrasound. Secondary outcomes included the Patient Specific Functional Scale (PSFS), Foot Function Index (FFI), Short Form 12 (SF-12), and EuroQoL 5D (EQ5D).

**Results:**

Significant reductions in NPRS scores at rest (2.98 to 1.39, *p* = 0.004) during activity (6.64 to 2.66, *p* ≤ .001), and during the first step in the morning (6.79 to 2.69, *p* ≤ .001) were observed. Plantar fascia thickness in the unilateral group also decreased significantly over time (affected side 5.34 mm to 3.70 mm, *p* ≤ 0.001). PSFS scores improved significantly, indicating reduced difficulty in performing activities. The FFI showed significant improvements in pain, disability, and activity subscales. Quality of life showed significant improvement in the physical component scores.

**Discussion:**

Dropout during follow-up, variability in therapist experience, missing data in patient-reported outcomes, and the lack of a standardized ultrasound examiner may have influenced reliability. Additionally, the intervention combined two treatments—(ortho)manual therapy and corrective insoles—making it difficult to isolate their individual effects. Despite these limitations, significant improvements in pain and plantar fascia thickness were observed as early as 1–2 weeks after the first treatment session, with clinically relevant changes sustained over 26 weeks. Due to the limitations, the results of this study should be interpreted with caution, but support (ortho)manual therapy as a promising treatment for PF. Further controlled studies are needed to confirm these findings.

**Conclusion:**

The results show pain reduction within 6–8 weeks with further improvement at follow up. The treatment has lasting effects, potentially preventing PF recurrence and other lower extremity issues. Ultrasound findings may alter the understanding of PF and tendon problems.

## Introduction

Plantar fasciitis (PF) is a common cause of heel pain. About 10% of the population will experience it at some point in their life and approximately 15% of all foot pathologies can be attributed to PF (Rhim et al., 2021). PF is experienced as very painful, persistent and restrictive for work and/or sports (Gorter & Louwerens, 2003). In the Netherlands, PF is even mentioned in several consecutive years as one of the most common occupational diseases of the lower extremity (Coronel Instituut voor Arbeid en Gezondheid, 2018). PF occurs most often in people between the ages of 40 and 60, in both non-active and active individuals. Running has the highest prevalence of 17.4% (Rhim et al., 2021).

Studies show a variety of risk factors; from decreased ankle dorsiflexion, overpronation, increased BMI, to a standing occupation (Yi et al., 2011; Hamstra-Wright et al., 2021). The applied therapies are just as diverse; medication, exercises, shockwave, insoles, injections, massage, mobilizations up to surgery and immobilization (Grim et al., 2019; Siriphorn & Eksakulka, 2020; Franettovich Smith et al., 2020; Bartold, 2004; Rhim et al., 2021). The systematic review by Rhim et al. (2021), which analysed 96 other systematic reviews related to PF, shows that corticosteroids can provide short-term pain relief (up to one month), especially when administered under ultrasound guidance. However, the benefits are often not long-lasting, and there is a risk of complications such as plantar fascia rupture. In the longer term (3 to 12 months), platelet-rich plasma (PRP) and extracorporeal shockwave therapy (ESWT) show better outcomes than corticosteroids. Mechanical interventions such as insoles and taping provide limited and short-term relief, while stretching, manual therapy, and laser therapy are more consistently associated with positive effects. A key issue across all these therapies is the variability in how they are applied; the effectiveness of PRP depends greatly on its composition, and there is no consensus on the optimal settings for ESWT regarding intensity, number of shocks, and treatment location, different kind of tapes are applied and manual therapy can be massage or more joint related therapy (Rhim et al., 2021). A systematic review and meta-analysis by Guimaraes et al. (2022), which also examined the effects of various interventions on pain in plantar fasciitis, found that shockwave therapy, orthoses, and corticosteroid injections are the most studied treatments in the literature. In this study, low-level laser therapy was the only intervention to show a moderate effect on pain, while the others showed only a low effect.

The quantity and diversity of these therapies show that there is not yet a “best” treatment or protocol. Hansen et al. (2018) performed a longitudinal study over 5–15 years and the results showed that on average, subjects had tried 3.8 different treatments and after an average follow-up time of 9.6 years, 46% of the 174 participants still suffered from symptoms. 54% subjects didn’t have any symptoms at the follow-up, but the symptoms has lasted a mean of 725 days/2 years. Poenaru, Badoiu & Ionescu (2021) also state that in the majority of patients, the complaints disappear as a result of natural course within 2 years, but given the pain and the debilitating nature of the complaints, only a few patients want to try the wait-and-see policy. The economic burden of PF in the United States in 2010 was stated at $284 million dollar (Tong & Furia, 2010). Enough reasons to find a therapy that is efficient, quick and long-term resistant.

The pathophysiology of PF is thought to involve excessive strain of the plantar fascia due to the repetitive microtrauma associated with persistent load-bearing, which eventually incites an inflammatory response and degenerative changes (Brachman et al., 2020). This degenerative change often leads to a thickening of the tendon plate, where a thickness above four mm in combination with the clinical findings, generally is used as the official diagnosis (Rajaonarison Ny Ony Narindra et al., 2019). The risk factors described earlier contribute to this mechanism by increasing the mechanical load beyond normal thresholds.

To positively influence the recovery time of tendons and ligaments, it is necessary to reduce the excessive strain (Cook & Purdam, 2009). Most therapies mainly address the symptoms or risk factors by aiming to reduce the excessive strain (sole therapy, stretching the calf and arch, strengthening the muscle) or by locally stimulating the tissue for recovery (shockwave, massage or injections) (Bartold, 2004). However, these therapies generally do not aim to address the underlying mechanism of why the excessive strain in the foot develops in the first place.

Sagittal Plane Biomechanics of Dananberg (2000) tries to give an explanation for this underlying cause of PF. In normal biomechanics, the distal and proximal aspects of the plantar fascia comes closer together at the moment of terminal stance, as a result of the windlass mechanism. For the windlass mechanism to work well, dorsiflexion of the ankle and metatarsophalangeal joints are needed. If these are limited, the midtarsal joints are the only site available in the foot for sagittal movement. If compensation in the sagittal plane is required, the distal and proximal aspects will instead move apart from each other as the midtarsal joint is forced to provide sagittal plane motion under the influence of the force transmitted from above. This causes the medial longitudinal arch to collapse and being stretched at the wrong moment, possibly causing the repetitive excessive strain, or mechanical overuse, of the plantar fascia (Dananberg, 2000; Donatelli, 1985). By removing the mechanical overuse, it could become possible to (re-)initiate the recovery process and allow the plantar fascia to heal and return to a thickness less than four mm (Cook & Purdam, 2009). Based on this explanation, manual therapy could be a potential treatment for removing the mechanical overuse by restoring the joint limitations. Recovery time will still be difficult to predict, tendons generally heal slowly and it’s unclear if overuse is the main and only reason of tissue impairment (Cook et al., 2016).

VoetPortaal is a Dutch orthomanual and podiatry clinic which has based their therapy for PF on this theoretical model of Sagittal Plane Biomechanics (Dananberg, 2000). The average treatment time takes 6–8 weeks and patients generally report improvements in pain, activities and quality of life. These observations suggests that (ortho)manual therapy may reduce excessive strain on the plantar fascia, potentially facilitating healing. The current study was designed as a single-arm cohort, following a larger group of patients of VoetPortaal with PF, to monitor and objectify the longitudinal course of PF and the changes in pain, function, quality of life and if these changes are accompanied by a change in thickness of the tendon plate. A single-arm design was chosen because the (ortho)manual therapy combined with the custom insoles, represents the clinic’s usual care. Additionally, since treatment was not free of charge, assigning patient to a no-treatment group would not have been ethical.

Based on the theoretical model of Sagittal Plane Biomechanics of Dananberg (2000) and the clinical observations, we hypothesize that if (ortho)manual therapy is applied to individuals with plantar fasciitis, this intervention will lead to a significant reduction in pain and tendon plate thickness, as well as improvements in physical functioning and quality of life, due to the restoration of joint mobility and the reduction of mechanical overuse of the plantar fascia. Also by monitoring the course over time, we can better understand which factors contribute to the progression or improvement of the condition and how we can optimize the care of patients with PF. It can also help to improve the knowledge about the recovery mechanism of the tendon plate and thereby the pathophysiology of plantar fasciitis.

Therefore the research question is “What changes over time are measured on pain, activities, quality of life and thickness of the tendon plate when (ortho)manual therapy of the foot is applied to people with plantar fasciitis and to what extend can these changes be attributed to the given therapy?”

## Materials & Methods

### Study design

This study was a prospective observational single-arm cohort study.

### Participants

Patients were consecutively recruited in a 22-month period from January 2021 until October 2022. Patients were screened for eligibility by the therapists of VoetPortaal at the first visit to the clinic. Inclusion criteria for study participation were: age over 18 years with plantar heel pain, suspected or diagnosed with calcaneal spur included. Patients with the following comorbidities were excluded from participation; Diabetics, CVA, MS and osteoarthritis in one or multiple foot or toe joints. Other exclusion criteria were; complaints caused by trauma, complaints paired with Achilles tendon pain, severe foot and/or lower extremity deformities, suspected tarsal tunnel syndrome, suspected stress fractures, suspected neurological involvement, infections or tumors in the painful foot, fat pad syndrome and use of non-steroidal anti-inflammatory drugs (NSAIDs).

Fifty participants were recruited for T1. 31 females and 19 males were included with an average age of 52.46 (SD ± 9.89). 21 subjects had a left affected side, 17 a right affected side and 12 were bilateral affected. The medial tuberosity of the calcaneus was the most reported painful location. Most of the subjects had complaints for a few months to six months. Various specialists and therapies have been visited previously. Baseline demographics and characteristics are reported in Table 1 of all 50 patients. The average number of treatments was 6.6. When additional treatments were given, they were mostly given between T5 and T6.

**Table 1:** Baseline Demographics, Measurements and Questionnaires at T1.

*N* = 50, Mean (SD) unless otherwise stated			
**Age, y**	52.46 (9.89)	**Pain history, No**	
**Sex, female, male, No**	31/19	Left/Right/ Bilateral	21/17/12
**Educational Level, No**		Course of plantar fascia	6
WO/Post Doc	22	Middle of calcaneus	12
HBO	15	Medial tuberosity on the calcaneus	20
MBO	9	Outer edge of the heel	7
High School	4	Combined	17
**Height**	176.90 (10.27)	**Duration of pain, No**	
**Weight, kg**	84.24 (15.72)	A few days	2
**BMI**	26.96 (4.78)	A few weeks	8
**Profession, No**		A few months	18
Standing	12	A halve year	6
Seated	26	Longer than a halve year	4
Combined	8	Longer than a year	12
None/Retired	4	**Measurements**	
**Self-Reported Comorbidities, No**	5	NPRS during rest (0–10)	2.96 (2.59)
**Other musculoskeletal pain in the past, No**	36 (Ankle = 5, Knee = 4, LBP = 10, Shoulder = 2, Combined = 4)	NPRS during activity (0–10)	6.44 (2.04)
**Other musculoskeletal pain** ** at first visit, No**	34 (Ankle = 3, Knee = 3, Hip = 1, LBP = 7, Combined = 10)	NPRS first step in the morning (0–10)	6.62 (2.38)
**Visited Specialists**		PSFS (0–30)	15.08 (6.27)
GP	27	**Questionnaires**	
Physical Therapist	21	DN4 (0–10)	2.80 (2.07)
Podiatrist	13	FFI pain (0–100), *N* = 49	65.55(20.91)
Orthopedist	3	FFI disability (0–100), *N* = 49	46.37 (23.58)
Other/None	15	FFI activity (0–100), *N* = 49	17.37 (16.01)
Combined specialists	18	FFI Total (04-100), *N* = 49	46.82 (19.27)
**Other interventions in the past, No**		SF 12 Physical health (0–100)	39.88 (10.01) USA diff. −10.16 (10.03)
Pain medication	11	SF 12 Mental health (0–100)	52.49 (8.42) USA diff. 8.50 (18.27)
Exercises	20	EQ5D score	0.80 (0.07)
Shockwave	14	EQ5D Health VAS score, *N* = 48	6.98 (1.83)
Dry Needling	2	**Physical Examination**	
Massage	10	Plantar fascia thickness affected side, mm	Left 0.54 (0.17), *N* = 19 Right 0.52 (0.13), *N* = 15
Manual Therapy	3	Plantar fascia thickness non affected side, mm	Left 0.38 (0.14), *N* = 18 Right 0.35 (0.05), *N* = 16
Corticosteroid injections	1	Plantar fascia thickness bilateral, mm	Left 0.44 (0.10), *N* = 12 Right 0.45 (0.10), *N* = 12
Taping	5	Limited Dorsiflexion (<10°) Subtalar Joint Right Affected Side	14/17
Custom insoles	15	Limited Dorsiflexion (<10°) Subtalar Joint Left affected side	17/21
Of the shelf soles	4	Limited Dorsiflexion (<10°) Subtalar Joint Bilateral	8/12
Gel heel insoles	16		
Strassburg sock	8		
Specific shoe wear	6		
Other	12		
Combined	24		

**Notes.**

SD standard deviation y years No number kg kilograms BMI body mass index LBP Low Back pain GP General Physician NPRS Numeric Pain Rating Scale PSFS Patient Specific Functional Scale DN4 Douleur Neuropathique FFI Foot Function Index SF 12 12 item Short Form Health Survey EQ5D EuroQol five dimension scale VAS Visual Analogue Scale mm millimeters

### Dropouts

**Figure 1:**
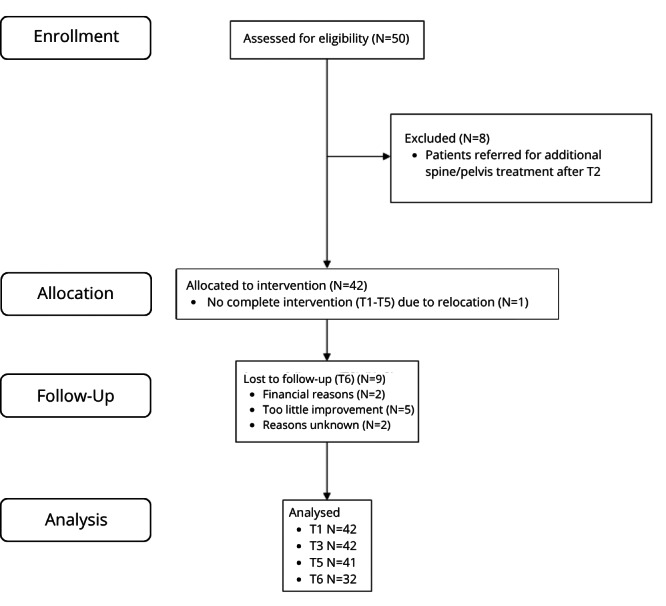
Flow diagram.

A total of 18 patients dropped out; 8 patients were excluded after T2 because of the extra spine treatment and were left out on any further analysis. 10 patients dropped out during the remainder of the study; one patient dropped out before T5 was completed due to relocation, nine patients didn’t complete T6; two patients did not want to return due to financial reasons, five patients dropped out due to too little improvement and choice for other interventions, two patients cancelled the appointment with no reason. See Fig. 1 for the flow diagram. Table 2 shows the baseline demographics and the main outcomes at T1 of the 32 patients who completed the study and the 10 dropouts. None of the outcome measures is significantly different between these two groups.

**Table 2:** Baseline demographics and outcomes of study population that completed the study and dropouts.

	Population completed the study *N* = 32. Mean (SD) unless otherwise stated	Dropped out population *N* = 10. Mean (SD) unless otherwise stated	Significance between groups. *p*-value
**Age. y**	52.47 (10.99)	53.40 (4.99)	0.81
**Sex. female. male. No**	22/10	4/6	
**Height**	174.72 (10.67)	180.80 (10.25)	0.12
**Weight. kg**	81.47 (15.08)	90.50 (14.23)	0.19
**BMI**	26.73 (4.71)	27.81 (4.54)	0.63
**Pain score at T1**			
NPRS during rest (0–10)	2.75 (2.51)	3.55 (3.05)	0.57
NPRS during activity (0–10)	6.44 (1.98)	7.27 (1.62)	0.30
NPRS first step in the morning (0–10)	6.81 (2.05)	6.73 (2.90)	0.78
PSFS (0–30)	14.69 (6.16)	17.82 (7.04)	0.25
**Questionnaires at T1**			
FFI pain (0–100)	65.00 (20.16)	66.80 (25.63)	0.53
FFI disability (0–100)	45.47 (21.00)	51.50 (30.27)	0.39
FFI activity (0–100)	16.56 (15.18)	20.40 (20.74)	0.87
FFI Total (0–100)	46.09 (17.70)	50.60 (24.56)	0.39
SF 12 Physical health (0–100)	39.62 (8.78)	38.62 (12.39)	0.72
	USA diff. −10.43 (8.82)	USA diff. −11.38 (12.39)	0.72
SF 12 Mental health (0–100)	52.97 (7.99)	51.45 (10.21)	0.63
	USA diff. 10.77 (19.48)	USA diff. 1.50. (10.20)	0.15
EQ5D score	0.81 (0.07)	0.79 (0.08)	0.70
EQ5D Health VAS score	7.09 (1.63) *N* = 30	6.91 (2.32)	0.91
**Pain score at T3**			
NPRS during rest (0–10)	2.84 (2.60)	3.00 (3.13)	0.53
NPRS during activity (0–10)	5.28 (2.30)	5.50 (2.68)	0.65
NPRS first step in the morning (0–10)	4.47 (3.01)	5.80 (2.74)	0.20
PSFS (0–30)	11.44 (5.95)	12.40 (6.17)	0.37
**Questionnaires at T3**			
FFI pain (0–100)	54.75 (18.86)	54.60 (24.99)	0.78
FFI disability (0–100)	36.22 (19.80)	39.20 (25.28)	0.70
FFI activity (0–100)	11.97 (9.32)	12.30 (12.44)	0.99
FFI Total (0–100)	37.34 (16.41)	38.80 (21.10)	0.87
**Pain score at T5**			
NPRS during rest (0–10)	2.56 (2.30)	2.44 (2.83). *N* = 9	0.79
NPRS during activity (0–10)	3.38 (2.89)	5.00 (2.35). *N* = 9	0.18
NPRS first step in the morning (0–10)	3.41 (2.71)	5.11 (2.62). *N* = 9	0.92
PSFS (0–30)	7.94 (5.70)	8.22 (6.22). *N* = 9	0.94
**Questionnaires at T5**			
FFI pain (0–100)	36.94 (23.38)	47.00 (20.26). *N* = 8	0.25
FFI disability (0–100)	20.56 (19.21)	32.88 (15.46). *N* = 8	0.10
FFI activity (0–100)	7.59 (10.46)	10.00 (10.69). *N* = 8	0.48
FFI Total (0–100)	23.16 (17.71)	33.00 (14.82). *N* = 8	0.15
SF 12 Physical health (0–100)	47.99 (8.04). *N* = 31	43.27 (9.26). *N* = 8	0.15
	USA diff. −1.98 (8.06)	USA diff. −6.73 (9.27)	0.15
SF 12 Mental health (0–100)	52.69 (7.24). *N* = 31	50.91 (11.90). *N* = 8	0.65
	USA diff. 2.70 (7.24)	USA diff. 0.90 (11.90)	0.65
EQ5D score	0.85 (0.08). *N* = 31	0.82 (0.10). *N* = 8	0.36
EQ5D Health VAS score	7.53 (1.40). *N* = 29	7.43 (1.08). *N* = 8	0.73

**Notes.**

SD standard deviation y years No number kg kilograms BMI body mass index NPRS Numeric Pain Rating Scale PSFS Patient Specific Functional Scale FFI Foot Function Index SF 12 12 item Short Form Health Survey EQ5D EuroQol five dimension scale VAS Visual Analogue Scale

### Ethical statement

The study was approved by the Medical Ethics Review Committee of the Amsterdam Medical Centre (AMC) on 19-03-2021, reference number W21_128 # 21.143. Patients were followed during the course of usual care. No control group was included. Given the exploratory nature of the study and its focus on monitoring changes over time within a representative patient population, a within-subject design was considered most appropriate. Additionally, ethical considerations played a role, as withholding potentially effective treatment from patients with chronic pain complaints would not have been justifiable. All patients were informed about the procedures involved in the study and signed an informed consent.

### Sample-size estimation

The sample-size calculation was performed by using a power sample-size calculator. The following parameters were used: 2-sided test, power of 0.80, alpha of .05, effect size of 0.80 based on the numeric pain rating scale (NPRS). The number of subjects according to this calculation was a minimum of 20 subjects. Because of possible drop-outs and to add more strength to the outcomes, 50 participants were recruited.

### Procedures

The protocol consisted of 6 visits to VoetPortaal. At baseline (T1, week 1) patients provided demographic information, medical history and previous treatments of PF. A physical examination was performed. When the patient was eligible and wanted to participate in the study, the rest of the protocol was followed; the NPRS and patient specific functional scale (PSFS) were asked out and recorded in the digital patient record. The following questionnaires were filled; Short Form 12 (SF-12), Foot Function Index (FFI), Douleur Neuropatique (DN4) and the EuroQoL 5d (EQ5D).

**Figure 2:**
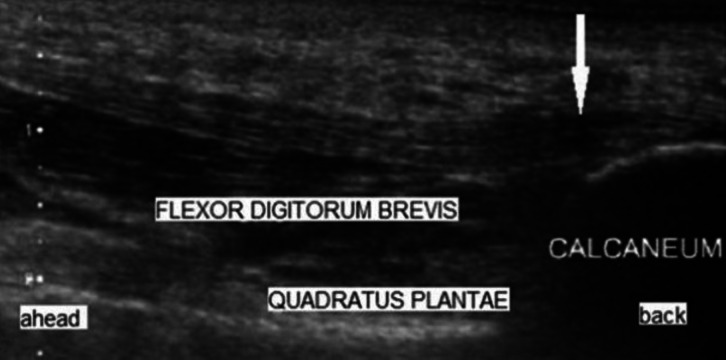
Ultrasound image of the plantar fascia illustrating the location at which thickness is measured (white arrow). Adapted from Rajaonarison Ny Ony Narindra et al. (2019).

At T1 the ultrasound and the 3D scan of the feet where made. The ultrasound was performed by the therapist. Patients where in sitting position on the treatment table. Both feet were measured regardless of whether there were complaints on one or two sides. The thickness of the plantar fascia was measured at the thickest portion between the base of the medial calcaneal tubercle and the outside line of the plantar fascia itself (see Fig. 2). A single measurement was taken for each foot at each measurement moment. The results of the ultrasound were recorded in the digital file. The measurement wasn’t used as a diagnostic tool, but evaluative to see the change in time.

At the second visit (T2, week 2) patients received the first treatment for one or both feet. Within this visit it was determined by the therapist if the patient needed additional spine/pelvis treatments. Patients who were referred for additional spine/pelvis treatment were excluded from the remainder of the study and analysis. The corrective insoles were given to the patient and checked for fitting.

The third visit (T3, week 3) consisted of taking the following questionnaires: NPRS, PSFS and the FFI. A brief physical examination of the gait pattern and foot function was done after which the second treatment took place. Insoles were checked for fitting and use. The fourth visit (T4, week 5) consisted of a brief physical examination of the gait pattern and foot function and the third treatment. In the fifth visit (T5, week 8) the following questionnaires were taken: NPRS, PSFS, SF-12, FFI and EQ-5D. The ultrasound for both feet was repeated. Treatment took place after a brief physical examination of the gait pattern and foot function.

The follow-up after 26 weeks (T6) consisted of the same measurements as at T5. T6 was purely for evaluation, so no treatment took place. Because the best care was paramount, patients were always able to call for additional appointments.

### Interventions

Patients received a combination of interventions; the (ortho)manual therapy and the corrective insoles. The (ortho)manual therapy consisted of techniques using a hammer and thrift and conventional hand-based manual therapy. Controlled, precise forces were applied to foot, ankle and fibula joints, to correct alignment. The therapy was performed on the joint impairments found in the physical examinations at T1. Since this can differ per patient, there was no specific treatment protocol that is performed in the same way for every patient. The different (ortho)manual techniques that were used are extensively described in Appendix S1. For the procedure of the corrective insoles see Appendix S2.

### Therapists

Seven different therapists participated in the study. All were trained in the same manual techniques prior to the start of the study. The therapists varied in their levels of professional experience. To ensure consistency, two calibration sessions were held during the study period to align on treatment protocols, research procedures, and ultrasound techniques. All therapists also performed the ultrasound examinations. A dedicated training session was provided to specifically instruct them in performing standardized ultrasound imaging of the heel.

### Materials

All patients received custom made insoles. The 3D-scan was made with the Paroscan 3DM of Paromed GmbH & Co. KG (Germany). Ultrasounds were made with the GE Venue 40 Ultrasound Machine of GE Ultraschall (Germany). Joint impairments were treated by (ortho)manual therapy. This therapy knows manipulations by hand, but also with the assistance of a special hammer and thrift, both with rubber endings.

### Outcome measures

#### Primary outcomes

The NPRS is a valid and reliable tool for measuring pain intensity (Shashua et al., 2015). The pain score at rest, during activities and during the first step in the morning were primary outcomes (0 as “no pain” and 10 as “very severe pain”). The minimal clinically important difference (MCID) varies between 1.7–2.0 points in musculoskeletal pain (Shashua et al., 2015).

The thickness of the plantar fascia is also a primary outcome. Rhim et al. (2021) have shown in their research that ultrasound can be a reliable imaging tool for measuring the plantar fascia thickness. In literature a thickness >4 mm is considered as an absolute plantar fasciitis. In asymptomatic subjects, values between 1.8 and 4.3 mm are reported (Rajaonarison Ny Ony Narindra et al., 2019).

#### Secondary outcomes

The secondary outcomes of this study consisted of the results of the PSFS, FFI, SF-12 and the EQ5D.

With the PSFS, patients are asked to define the 1–3 activities that are most important. Each activity was then scored on a scale from 0–10, (0 as “no problem with performing the activity” and 10 as “not possible to perform the activity”). For a total PSFS score the item scores of the activities are added up. The higher the total score, the more problems experienced when performing the activities. The MCID for the PSFS has been reported in studies about knee dysfunction, cervical radiculopathy, and chronic low back pain, and varied between 2.0 and 3.0 points per activity (Kowalchuk Horn et al., 2012).

The Foot Function Index (FFI) is a questionnaire that assesses multiple dimensions of foot function. It consists of 23 items divided into 3 subscales; pain, disability, and activity limitation and has been widely used by clinicians and investigators to measure pain and disability in a variety of foot and ankle disorders (Budiman-Mak et al., 2013). The MCID for pain is −12, −7 for disability and −7 for total foot function index. The sub scale activity limitation has a MCID that came out as 0 (−0.5).

The SF-12 is an appropriate measure to capture the quality of life of patients. Scores range from 0 to 100. A higher score indicates a better physical and mental health functioning. A score of 50 or less on the PCS-12 is a cut-off to determine a physical condition. A score of 42 or less on the MCS-12 may indicate a clinical depression (Ware, 2002). The EQ5D measures five dimensions that are all related to quality of life: mobility, self-care, activities, pain and anxiety. The EQ VAS records the patient’s self-rated health on a vertical visual analogue scale.

### Statistical analysis

All data were entered and analyzed in the statistical package for the social sciences (SPSS Inc, Chicago, IL), version 29.0 for Windows. Data analysis procedures were performed in accordance with previously described methods (Van Egmond, 2020). All statistical tests were two-sided, and statistical significance was defined as an alpha level of 0.05. Baseline characteristics were summarized using descriptive statistics. Discrete variables were presented as counts and percentages, ordinal variables as medians with interquartile ranges (P25–P75), and continuous variables as means with standard deviations. In case of non-normally distributed data, continuous variables were reported as medians with interquartile ranges. Linear mixed-effects model with random intercepts and random slopes for time at the patient level were used to examine changes over time in pain, plantar fascia thickness, function, participation, and quality of life. Time was included as a fixed effect (categorical). An unstructured covariance matrix was used. Standardized effects were calculated by dividing the fixed-effect estimates by the baseline standard deviation of the specific variable.

## Results

### Primary outcomes

Numeric pain rating scale.

### NPRS in rest

The NPRS at rest (see Table 3) at T6 was significantly lower than at T1 (*p* = 0.004, 95% CI [−2.73 to −0.55]), T3 (*p* = 0.002, 95% CI [−2.43 to −0.62]) and T5 (*p* = 0.006, 95% CI [−2.04 to −0.37]). The average decrease from T1 to T6 was 1.59 points (53.36%). The MCID of the NPRS varies between 1.7–2.0. The average decrease was close to the MCID. Patients indicated a fairly low score at T1 for the NPRS at rest, making it more difficult to achieve a decrease of 1.7–2.0.

**Table 3:** Numeric Pain Rating Scale (NPRS), Patient Specific Functional Scale (PSFS), Foot Function Index (FFI) and Quality of Life (QoL) over time analysed using linear mixed models.

1	T1 Mean (SD)	T3 Mean (SD)	T5 Mean (SD)	T6 Mean (SD)	*p*-value	95% CI	*β* (Estimate)	SE	Std. effect
NPRS in rest (1–10)	2.98 (2.67) *N* = 42	2.88 (2.69) *N* = 42	2.54 (2.39) *N* = 41	1.39 (2.20) *N* = 32	T3 - T1 *p* = 0.829 T5 - T1 *p* = 0.429 T6 - T1 *p* = 0.004 T5 - T3 *p* = 0.428 T6 - T3 *p* = 0.002 T6 - T5 *p* = 0.006	(−0.98, 0.79) (−1.45, 0.63) (−2.73, −0.55) (−1.09, 0.47) (−2.43, −0.62) (−2.04, −0.37)	−0.10 −0.41 −1.64 −0.31 −1.53 −1.20	0.44 0.51 0.54 0.38 0.45 0.41	−0.04 −0.15 −0.04 −0.12 −0.57 −0.50
NPRS during activity (1–10)	6.64 (1.94) *N* = 42	5.33 (2.37) *N* = 42	3.73 (2.84) *N* = 41	2.66 (3.09) *N* = 32	T3 - T1 *p* = 0.003 T5 - T1 *p* = <.001 T6 - T1 *p* = <.001 T5 - T3 *p* = <.001 T6 - T3 *p* = <.001 T6 - T5 *p* = 0.048	(−2.13, −0.49) (−3.75, −1.93) (−5.18, −2.93) (−2.42, −0.68) (−3.86, −1.66) (−2.29, −0.01)	−1.31 −2.84 −4.06 −1.55 −2.76 −1.15	0.41 0.45 0.55 0.43 0.54 0.56	−0.68 −1.46 −2.09 −0.65 −1.16 −0.40
NPRS 1st step in the morning (0–10)	6.79 (2.29) *N* = 42	4.79 (2.97) *N* = 42	3.78 (2.75) *N* = 41	2.69 (3.16) *N* = 32	T3 - T1 *p* = <.001 T5 - T1 *p* = <.001 T6 - T1 *p* = <.001 T5 - T3 *p* = 0.042 T6 - T3 *p* = 0.002 T6 - T5 *p* = 0.033	(−2.84, −1.15) (−3.87, −2.06) (−4.92, −2.89) (−1.91, −0.03) (−2.99, −0.73) (−1.77, −0.08)	−2.00 −2.97 −3.91 −0.97 −1.86 −0.93	0.42 0.45 0.50 0.46 0.56 0.43	−0.87 −1.30 −1.71 −0.33 −0.63 −0.34
PSFP (0–10)	15.29 (6.40) *N* = 42	11.67 (5.94) *N* = 42	8.00 (5.74) *N* = 41	5.39 (5.96) *N* = 32	T3 - T1 *p* = <.001 T5 - T1 *p* = <.001 T6 - T1 *p* = <.001 T5 - T3 *p* = <.001 T6 - T3 *p* = <.001 T6 - T5 *p* = 0.015	(−5.38, −1.86) (−9.45, −5.06) (−11.99, −7.36) (−5.22, −2.04) (−8.10, −4.13) (−4.58, −0.54)	−3.62 −7.25 −9.68 −3.63 −6.12 −2.56	0.87 1.09 1.14 0.79 0.98 0.99	−0.57 −1.13 −1.51 −0.61 −1.03 −0.45
FFI Pain	65.43 (21.26) *N* = 42	54.71 (20.15) *N* = 42	38.95 (22.91) *N* = 40	23.53 (23.48) *N* = 30	T3 - T1 *p* = <.001 T5 - T1 *p* = <.001 T6 - T1 *p* = <.001 T5 - T3 *p* = <.001 T6 - T3 *p* = <.001 T6 - T5 *p* = <.001	(−15.35, −6.07) (−32.67, −18.62) (−49.46, −32.38) (−21.13, −8.79) (−37.62, −22.71) (−21.82, −6.63)	−10.71 −25.65 −40.92 −14.96 −30.16 −14.22	2.30 3.48 4.21 3.05 3.65 3.73	−0.50 −1.21 −1.92 −0.74 −1.50 −0.62
FFI Disability	46.90 (23.26) *N* = 42	36.93 (20.94) *N* = 42	23.03 (19.00) *N* = 40	13.10 (18.07) *N* = 30	T3 - T1 *p* = <.001 T5 - T1 *p* = <.001 T6 - T1 *p* = <.001 T5 - T3 *p* = <.001 T6 - T3 *p* = <.001 T6 - T5 *p* = 0.014	(−15.46, −4.49) (−29.67, −16.29) (−41.52, −24.58) (−19.65, −6.59) (−29.58, −16.52) (−16.12, −1.99)	−9.98 −22.98 −33.05 −13.12 −23.04 −9.06	2.71 3.31 4.18 3.23 3.21 3.47	−0.43 −0.99 −1.42 −0.63 −1.10 −0.48
FFI Activity	17.48 (16.48) *N* = 42	12.05 (9.98) *N* = 42	8.07 (10.42) *N* = 40	5.77 (11.05) *N* = 30	T3 - T1 *p* = 0.043 T5 - T1 *p* = <.001 T6 - T1 *p* = <.001 T5 - T3 *p* = 0.009 T6 - T3 *p* = 0.002 T6 - T5 *p* = 0.309	(−10.67, −0.19) (−13.44, −4.42) (−18.31, −5.70) (−5.97, −0.90) (−10.65, −2.61) (−6.88, 2.25)	−5.43 −8.93 -12.00 −3.66 −6.31 −2.32	2.59 2.24 3.12 1.75 2.31 2.24	−0.33 −0.54 −0.73 −0.37 −0.63 −0.22
FFI Total	47.17 (19.32) *N* = 42	37.69 (17.37) *N* = 42	25.13 (17.45) *N* = 40	14.83 (17.63) *N* = 30	T3 - T1 *p* = <.001 T5 - T1 *p* = <.001 T6 - T1 *p* = <.001 T5 - T3 *p* = <.001 T6 - T3 *p* = <.001 T6 - T5 *p* = 0.005	(−13.87, −5.09) (−26.72, −15.65) (−39.26, −24.31) (−16.99, −6.52) (−28.17, −16.26) (−15.88, −3.05)	−9.48 −21.18 −31.78 −11.76 −22.21 −9.46	2.17 2.73 3.68 2.59 2.92 3.15	−0.49 −1.09 −1.64 −0.68 −1.28 −0.54
SF-12 PCS	38.00 ( 6.47) *N* = 42		47.26 (7.85) *N* = 39	48.73 (7.66) *N* = 30	T5 - T1 *p* = <.001 T6 - T1 *p* = <.001 T6 - T5 *p* = 0.202	(4.77, 10.37) (6.63, 13.05) (−1.10, 5.02)	7.57 9.84 1.96	1.38 1.57 1.50	1.17 1.52 0.30
SF-12 MCS	51.95 (8.92) *N* = 42		51.86 (7.97) *N* = 39	51.51 (6.63) *N* = 30	T5 - T1 *p* = 0.877 T6 - T1 *p* = 0.565 T6 - T5 *p* = 0.767	(−2.42, 2.07) (−2.83, 1.57) (−2.59, 1.93)	−0.17 −0.63 −0.33	1.11 1.08 1.10	−0.02 −0.07 −0.04
EQ5D VAS	6.71 (1.68) *N* = 42		7.34 (1.52) *N* = 37	7.20 (1.51) *N* = 28	T5 - T1 *p* = 1.000 T6 - T1 *p* = 1.000 T6 - T5 *p* = 0.414	– – (−0.83, 0.35)	– – −0.24	– – 0.29	– – −0.14
EQ5D index	0.79 (0.06) *N* = 42		0.84 (0.08) *N* = 39	0.88 (0.09) *N* = 28	T5 - T1 *p* = 0.002 T6 - T1 *p* = <.001 T6 - T5 *p* = 0.075	(0.15, 0.06) (0.03, 0.11) (0.00, 0.07)	0.04 0.07 0.03	0.01 0.02 0.02	0.66 1.16 0.50

**Notes.**

SD standard deviaton CI Confidence Interval SE Standard Error Std. Effect Standardized effect N number of participants analysed

Standardized effects were calculated by dividing the fixed-effect estimates by the baseline standard deviation of the specific variable.

### NPRS during activity

The NPRS score during activity (see Table 3) showed a significant decrease between T3 and T1 (*p* = 0.003, 95% CI [−2.13 to −0.49]), T5 and T1 (*p* ≤ .001, 95% CI [−3.75 to −1.93]) and T6 and T1 (*p* ≤ .001, 95% CI [−5.18 to −2.93]). T5 was also significantly lower than T3 (*p* ≤ .001, 95% CI [−2.42 to −0.68]) and T6 was significantly lower than T5 (*p* = 0.048, 95% CI [−2.29 to −0.01]). This means that there was a significant decrease in pain score between all measurement moments. The largest reduction was seen between T3 and T5; 1.60 points. The MCID was met at T5 compared to T1, an average of 2.91 points decrease in pain. At T6 there was an average decrease in pain of 3.98 (59.94%) points compared to T.

### NPRS during 1st step in the morning

Pain decreased significant between measurement moments T3-T1 (*p* ≤ .001, 95% CI [−2.84 to −1.15]), T5-T1 (*p* ≤ .001, 95% CI [−3.87 to −2.06]) and T6-T1 (*p* ≤ .001, 95% CI [4.92 to −2.89]) and there was a significant decrease in pain between T5-T3 (*p* = 0.042, 95% CI [−1.91 to −0.03]), T6-T3 (*p* = 0.002, 95% CI [−2.99 to −0.73]) and T6-T5 (*p* = 0.033, 95% CI [−1.77 to −0.08]) (see Table 3); this means a significant decrease was found between each measurement moment. With an average decrease of 2.0 points between T1 and T3, the MCID was already met after 1 treatment. The average decrease in pain between T1 and T6 was 4.10 points (60.38%), well above the MCID.

### Ultrasound

For the ultrasound results, two groups were analyzed: one group with unilateral symptoms and one group with bilateral symptoms. The T1 values show that the affected side in the unilateral group and both sides in the bilateral group had a thickness greater than four mm, indicating that the diagnosis of plantar fasciitis was appropriate. The ultrasound results are presented in Table 4.

**Table 4:** Ultrasound over time analysed using linear mixed models.

		T1 Mean (SD), mm	T5 Mean (SD), mm	T6 Mean (SD), mm	*p*-value	95% CI	*β* (Estimate)	SE	Std. effect
Unilateral affected	Affected side	5.34 (1.61) *N* = 29	4.44 (1.30) *N* = 31	3.70 (0.86) *N* = 26	T5 - T1 *p* = <0.001 T6 - T1 *p* = <0.001 T6 - T5 *p* = 0.009	(−0.14, −0.05) (−0.22, −0.11) (−0.11, −0.02)	−0.10 −0.16 −0.06	0.02 0.03 0.02	−0.06 −0.10 −0.05
	Unaffected side	3.64 (1.13) *N* = 28	3.65 (0.86) *N* = 30	3.32 (0.51) *N* = 26	T5 - T1 *p* = 0.807 T6 - T1 *p* = 0.225 T6 - T5 *p* = –	(−0.06, 0.05) (−0.10, 0.02) –	−0.01 −0.04 –	0.03 0.03 –	0.00 0.04 –
Billateral affected	Left	4.49 (1.07) *N* = 10	3.71 (1.13) *N* = 9	3.47 (0.92) *N* = 6	T5 - T1 *p* = 0.009 T6 - T1 *p* = 0.016 T6 - T5 *p* = 0.606	(−1.20, −0.19) (−1.41, −0.16) (−1.00, 0.63)	−0.70 −0.79 −0.18	0.24 0.30 0.35	−0.65 −0.73 −0.16
	Right	4.41 (0.89) *N* = 10	03.67 (0.60) *N* = 9	4.18 (1.59) *N* = 6	T5 - T1 *p* = 0.086 T6 - T1 *p* = 0.734 T6 - T5 *p* = 0.329	(−1.60, 0.11) (−1.12, 0.80) (−0.65, 1.81)	−0.74 −0.16 0.58	0.41 0.47 0.57	−0.83 −0.18 0.97

**Notes.**

SD standard deviaton CI Confidence Interval SE Standard Error Std. Effect Standardized effect N number of participants analysed

Standardized effects were calculated by dividing the fixed-effect estimates by the baseline standard deviation of the specific variable.

### Affected side *versus* unaffected side

The affected side showed a decrease in thickness over time. Both T5 (*p* < 0.001; 95% CI [−0.14 to −0.05]) and T6 (*p* < 0.001; 95% CI [−0.22 to −0.11]) were significantly lower than T1. In addition, a significant decrease in thickness was observed between T5 and T6 (*p* = 0.009; 95% CI [−0.11 to −0.02]). The overall decrease over time was 30.71%. The unaffected side showed no significant changes.

### Bilateral affected

It is striking that the average thickness of the plantar fascia at T1 was less thick than in the groups that are unilaterally affected. It is also noticeable that the thickness of the left and right were almost the same. In time, the left side followed the same pattern as the unilateral affected groups. There was a significant decrease in thickness between T5 and T1 (*p* = 0.009, 95% CI [−1.20 to −0.19]) and between T6 and T1 (*p* = 0.016, 95% CI [−1.41 to −0.16]). The right side showed no significant differences.

### Secondary outcomes

Since PF is seen as an injury that can significantly limit work, sports and other activities, the hypothesis was that if pain would decrease, the level of activities would increase and if people would be able to participate more in society again, the quality of life would increase.

### Patient specific functional scale

The outcomes of the PSFS are in Table 3. With the PSFS, the lower the score, the less difficulty one has in carrying out the activities. The MCID varies between 2.0 and 3.0 points per activity. In this case the scores are added up, so a MCID lies within 6.0–9.0 points in total. In the study group there was a significant difference between T3-T1 (*p* ≤ .001, 95% CI [−5.38 to −1.86]), T5-T1 (*p* ≤ .001, 95% CI [−9.45 to −5.06]) and T6-T1 (*p* ≤ .001, 95% CI [−11.99 to −7.36]). There was a significant difference between T5-T3 (*p* ≤ .001, 95% CI [−5.22 to −2.04]) and T6-T3 (*p* ≤ .001, 95% CI [−8.10 to −4.13]). Also, T6 was significantly different from T5 (*p* = 0.015, 95% CI [−4.58 to −0.54]). The MCID was reached from measurement moment T5 and on average an improvement of 64.75% in time. Patients were able to perform their activities with noticeable less difficulty in 6–8 weeks and after 26 weeks this was even better.

### Foot function index

The FFI (see Table 3) was one of the questionnaires which has been filled in by the patients. The data of the FFI pain showed significant differences between T1-T3 (*p* ≤ .001, 95% CI [−15.35 to −6.07]), T1-T5 (*p* ≤ .001, 95% CI [−32.67 to −18.62]), T1-T6 (*p* ≤ .001, 95% CI [−49.46 to −32.38]), T3-T5 (*p* = <.001, 95% CI [21.13 to −8.79]), T3-T6 (*p* ≤ .001, 95% CI [−37.62 to −22.71]), and T5-T6 (*p* ≤ .001, 95% CI [21.82 to −6.63]). The MCID of −12 points was reached at T5. From T1 to T6 there was an average of 64.04% decrease in pain.

For the subscale FFI disability there was a significant difference between T1-T3 (*p* ≤ .001, 95% CI [−15.46, −4.49]), T1-T5 (*p* ≤ .001, 95% CI [−29.67, −16.29]), T1-T6 (*p* ≤ .001, 95% CI [−41.52, −24.58]), T3-T5 (*p* ≤ .001, 95% CI [−19.65, −6.59]), T3-T6 (*p* ≤ .001, 95% CI [−29.58, −16.52]) and T5-T6 (*p* = 0.014, 95% CI [−16.12 to −1.99]). The MCID of −7 was reached at T3. From T1 to T6 there was an average of 72.07% decrease in disability.

The FFI activity showed a significant difference between T1-T3 (*p* = 0.043, 95% CI [−10.67 to −0.19]), T1-T5 (*p* ≤ .001, 95% CI [−13.44 to −4.42]), T1-T6 (*p* ≤ .000, 95% CI [−18.31 to −5.70]), T3-T5 (*p* = 0.009, 95% CI [−5.97 to −0.90]), T3-T6 (*p* = 0.002, 95% CI [−10.65 to −2.61]). The MCID of −0.5 was easily reached at T3. The average change between T1 and T6 was 66.99%.

The FFI total is a calculation of all the previous sub scales. It is not surprising that this data followed the same pattern as the subscales. There was a significant difference between T1-T3 (*p* = <.001, 95% CI [−13.87 to −5.09]), T1-T5 (*p* ≤ .001, 95% CI [−26.72 to −15.65]), T1-T6 (*p* ≤ .001, 95% CI [−39.26 to −24.31]), T3-T5 (*p* ≤ .001, 95% CI [−16.99 to −6.52]), T3-T6 (*p* ≤ .001, 95% CI [−28.17 to −16.26]) and T5-T6 (*p* = 0.005, 95% CI [−15.88 to −3.05]). To reach the MCID, a decrease of −7 points was necessary, which was easily met at T3. The average change between T1 and T6 was 68.56%.

### Quality of life

QoL is measured at T1, T5 and T6. T3 was skipped as it was expected that not much could change on this domain within an average of 3 weeks.

Table 3 shows the results of the SF-12, a mental component score (MCS) and a physical component score (PCS). 50 points are assumed as average for a population. The study group started with a relative low score at T1 for the physical component in comparison with the 50-point average, which indicates the presence of a physical condition. There was a significant increase at T5 (*p* ≤ .001, 95% CI [4.77–10.37]) and T6 (*p* ≤ .001, 95% CI [6.63–13.05]) relative to T1 on the physical component. The score of T6 was still somewhat higher than that of T5, but there was no significant difference. The most gains were therefore achieved in the first 6–8 weeks. At the mental component, the scores already were higher than the average of 50 points at T1. There were no significant differences between measuring moments.

The EQ5D VAS (see Table 3) indicates someone’s own perceptive of his/her health status with 10.0 indicating the best health status. For the study group there was a slight increase in VAS score over time, but there were no significant differences found.

The EQ5D index (see Table 3) is formed by answering a number of questions about health. The maximum score of 1 for the EQ5D index indicates the best health state. There was an increase in health status over time and T5 (*p* = 0.002, 95% CI [0.15–0.06]) and T6 (*p* ≤ .001, 95% CI [0.03–0.11]) were significantly different from T1.

## Discussion

The results of the primary outcome measures, NPRS and thickness of the tendon plate, showed significant improvements over time. T3 was in the third week of the study protocol and only one (ortho)manual therapy session had taken place and the insoles were worn for 1–2 weeks. The minimal clinical important difference (MCID) of the NPRS 1st step in the morning was reached at T3, the NPRS during activity was met at T5. The results of the ultrasound showed a significant decrease at T5. The secondary outcome measures showed the same pattern; within 1–2 weeks a significant improvement and the MCID was met at T3 or T5. These changes occurred within the time span of 6–8 weeks and with a couple of treatments.

For NPRS at rest, the average decrease from T1 to T6 was 1.59 points (53.36%), just below the MCID of 1.7–2.0. The 95% confidence interval (CI [−2.73 to −0.55]) indicates that while some participants experienced clinically meaningful improvement, others may have shown smaller changes, potentially due to low baseline scores. NPRS during activity showed a more consistent decrease, with an average reduction of 2.91 points at T5 compared to T1 (95% CI [−3.75 to −1.93]). The largest changes were observed between T3 and T5. Pain with the first step in the morning also showed a clinically meaningful decrease of 2.0 points between T1 and T3 (95% CI [−2.84 to −1.15]), suggesting that even a single session of (ortho)manual therapy combined with insoles may be associated with fast improvements in morning pain.

Ultrasound measurements of tendon thickness showed significant decreases over time, (T5 *vs* T1: *p* ≤ 0.001, 95% CI [−0.14 to −0.05]; T6 *vs* T1: *p* ≤ 0.001, 95% CI [−0.22 to −0.11]), with an overall decrease of 30,71%. At T6, tendon plate had decreased below four mm, which is below the diagnostic threshold for plantar fasciitis. In participants with bilateral symptoms, the left side followed a similar trend, whereas the right side did not show significant changes. These findings suggest that improvements in tendon thickness may can occur within a short period, although responses were variable across all patients.

Secondary outcome measures showed statistically significant and clinically meaningful improvements. For example, the FFI total score decreased by 68.56% between T1 and T6, surpassing the MCID of −7 points. Confidence intervals for these measures indicate consistent improvements across the majority of participants, supporting the notion that reductions in pain may be accompanied by enhanced function and participation.

The current prevailing conservative treatments for PF include shockwave therapy and the use of insoles, whether custom-made or not. Studies focusing on the efficacy of insoles as a standalone treatment show little to no effect in the first 3 months of the intervention. If effects are found, they are found in the long term, after about six months (Koc Jr et al., 2023). Ribeiro & Joäo (2022) compared minimalistic shoes and shoes with an insole with each other. After three months both groups experienced a decrease in pain, in favour of the insole group. These changes after three months are roughly similar in magnitude to the improvements observed in our study after six weeks. Rasenberg et al. (2021) found no difference in pain and function after 12 weeks in their study on the effect of insoles of PF. It is important to note that insoles or shoes do not directly alter the hypothesized etiology of excessive strain in PF and PF symptoms often come back when these interventions are discontinued (Koc Jr et al., 2023). Shockwave is widely used by physiotherapists as a treatment for PF with, in general, positive results within 3 months, but due to variations in parameters, session frequencies and the presence of calcifications, it is difficult to determine a golden standard (Rhim et al., 2021). Tezel et al. (2020) conducted a study with 84 subjects with plantar fasciitis, evaluating the effects of shockwave therapy *versus* kinesio taping on pain levels, functional status, quality of life and tendon plate thickness after a 6-week period. Shockwave was applied weekly with 2,000 shots at a frequency of six times per second and an energy intensity level of 0.2 ml/mm2 focused shockwaves. After 6 weeks there was a significant decrease in pain and a increase in functional status and quality of life. The tendon plate had become thinner, but this was not significant. Wu et al. (2020) studied 22 participants over a time span of 12 months and measured pain and tendon plate thickness. The intervention consisted of 3 following weeks with 1 session of shockwave per week. 3000 shock waves per session of 0.08–0.2 mJ/mm2 were applied. On the outcome of pain there was a significant decrease after 1 week. The thickness of the plantar fascia was significantly thinner after 12 months.

Shockwave therapy and insoles may improve symptoms, but their effect on the underlying excessive strain remains unclear, and recurrence is possible when discontinued. The goal of (ortho)manual therapy on joint blockages is aimed to influence the cause of the excessive strain on the plantar fascia and restoration of the windlass mechanism, creating a more favourable environment for tendon recovery. With an optimal environment, tendon tissue can recover within 3–6 weeks (Cook & Purdam, 2009). The thickness of the plantar fascia can be an objective indicator of treatment effectiveness, or recovery, whereas a thinner plantar fascia is associated with less symptoms and lower patient-reported pain scores (Latt et al., 2020). Our ultrasound and pain data suggest that the intervention of orthomanual therapy in combination with the correction insole may support this favourable environment.

Our findings on pain, function and quality of life are in line with a recent heel pain guideline from the American Physical Therapy Association (Koc Jr et al., 2023). In this guideline there is a recommendation for manual therapy as part of a multimodal treatment approach based on high levels of evidence. One of the manual therapy-focused studies referenced in this guideline is from Kashif et al. (2021). They compared 2 groups in their study; the intervention group received subtalar mobilization using the mulligan technique, stretching and taping. The control group received therapeutic ultrasound, stretching and taping. The outcome measures were pain and function. Both groups showed progress, but the progress in the intervention group with the mobilization was better over the 3-week intervention time. Grim et al. (2019) compared three groups with each other: manual therapy, custom-made insoles and a combined therapy group. The manual therapy consisted of treating the foot and spine in a standardized order. Pain, function and quality of life were measured. All groups had a reduction in pain and an improvement in function and quality of life, but the effects were greater in the isolated manual therapy group over the 3-month intervention period. These studies indicate that manual therapy may produce benefits in a relatively short period, but direct comparisons across interventions should be interpreted cautiously.

### Strengths and limitations

Due to the regular care approach, participants were not obliged to participate in the entire process and due to, for example, vacation, work, illness or financial reasons, the trajectory of some patients were not as strict as was drawn up in the protocol. A total of 10 patients dropped out; 1 patient dropped out before T5 was completed due to relocation. 9 patients didn’t complete the follow up; 2 patients did not want to return due to financial reasons, 6 patients dropped out due to too little improvement and choice for other interventions, 2 patients cancelled the appointment with no reason. The dropout of patients with minimal progress could have biased the T6 results to appear more favorable. However, at baseline (see Table 2) and up to T5, the dropouts did not differ significantly from the study group that completed T6. The influence of the dropouts on the course over time is therefore expected to be limited.

The NPRS and PSFS were outcome measures which were questioned by the therapist at the beginning of each visit and therefore there is not much missing data. The FFI, SRLS, SF-12 and EQ5D were questionnaires which were filled out in the waiting area and were submitted to the therapist. The random missing data was due to incorrect completion and because some questionnaires were not handed in. Linear mixed models were used to analyze the longitudinal data, which allows inclusion of participants with incomplete data at some time points, reducing loss of information and preserving statistical power, however missing data could still have affected the results.

Orthomanual therapy of the feet has been provided by various therapists. While a basic protocol was used for the assessment and techniques, every therapist will work different. It was preferred to have 1 participant seen by 1 therapist, but usual care did not always allow this. The level of experience of the therapist may have influenced outcomes, but these effects were not formally quantified.

Rhim et al. (2021) confirmed that ultrasound could be an accurate and reliable imaging tool for diagnosing and evaluating plantar fascia, but with the recommendation that the ultrasound should be made by an experienced therapist. The ultrasound in this study was made by the treating therapist at the time of the visit and they were not experienced sonographers. An ultrasound protocol was used here, but variability in measurements may arise due to inexperience and technique. Only one measurement per foot was taken each time, and neither intra- nor inter-rater reliability was assessed. Additionally, PF thickness was recorded and extracted from patient files, rather than stored images, limiting verification. With the ultrasound data, the groups become somewhat small because of splitting the participants in unilateral or bilateral sided complaints. These factors reduce the reliability of the ultrasound results, and conclusions should be interpreted cautiously, although general patterns were observed.

It was decided to follow the regular therapy over time as offered by VoetPortaal. This therapy combines 2 forms of therapy, namely treating joint blockages in the foot and wearing the corrective insole. It is not immediately clear which component contributed more to the observed changes. During the 2nd visit (T2), patients receive the first (ortho)manual treatment and their corrective insoles. The 3rd visit takes place the following week (T3) and various outcomes are measured. After this one week, significantly less pain is experienced and people feel less limited in activities. With the various literature on sole therapy in mind, where little to no effect is achieved (Rasenberg et al., 2021; Rasenberg et al., 2018) or where the effect is in any case only visible after a few weeks, it is plausible that (ortho)manual therapy had a notable impact. Due to the single-arm design, causal claims regarding the effect of the treatment cannot be made. Natural recovery through time, placebo effects or other confounders may have contributed to the observed outcomes. To fully break this down, the two therapies would have to be pitted against each other in four groups with a random assignment (insoles alone, (ortho)manual therapy alone and a combination of these therapies), together with a control group.

### Clinical implications

For the professional field, (ortho)manual therapy of the foot may represent a valuable and accessible treatment option capable of producing early improvements. For physical therapists or podiatrist, the (ortho)manual techniques can be learned with appropriate training. Ultrasound findings may change the view on the existing etiology. For example the much mentioned overpronation as a cause of the excessive strain whereas the overpronation is not the problem, but the joint blockages which are causing the foot to overpronate. Creating a more optimal environment for rapid tendon recovery. Not all patients responded optimally; dropouts due to insufficient improvement indicate that this therapy may not be suitable for everyone. In order to better select (and therefore advise) the patients that will mostly benefit from this therapy, further research is needed to specify if confounders, such as duration of the complaint, gender or weight, have influence on the results.

## Conclusions

Summarizing, it can be said that the pain (NPRS pain and FFI pain) decreases in time from T1 till T6. The most notable improvements occurred between T1 and T5 with 95% confidence intervals generally showing clear negative ranges, confirming consistent and statistically reliable reductions. These data suggests that clinically meaningful improvements may occur within 6–8 weeks, potentially faster than the conventional wait-and-see approach. In most groups, the MCID is met between T5 and T1, indicating there is also a noticeable difference for the patient in this timespan. Continued improvements between T5 and T6, suggest that effects may persist over time. Within activities and participation, the same trends were observed. The hypothesis for the quality of life is partially validated, mostly on the physical component. Ultrasound outcomes indicate that tendon recovery may be possible within 6–8 weeks, and perhaps further up to 26 weeks. This therapy appears to act not only on the symptoms, but may contribute to more sustainable changes. Given the many limitations, no direct causal link can be established between the therapy and the outcomes. However, the results highlight the potential of the intervention and warrant further investigation. To really determine if the therapy is effective and which part is effective, a suggestion for further research would be a factorial randomized controlled trial; 1 group receiving (ortho)manual therapy alone, 1 group receiving only the corrective insoles, 1 group receiving the combination of (ortho)manual therapy and corrective insoles and 1 control group receiving no intervention.

## Supplemental Information

10.7717/peerj.21280/supp-1 Supplemental Information 1 (Ortho)Manual therapy techniques

10.7717/peerj.21280/supp-2 Supplemental Information 2 Corrective insoles

10.7717/peerj.21280/supp-3 Supplemental Information 3 Code Book English-language codebook

10.7717/peerj.21280/supp-4 Supplemental Information 4 Strobe checklist

10.7717/peerj.21280/supp-5 Supplemental Information 5 Data from all subjects recoded over time for outcome measurements Numeric Pain Rating Scale, Patient Specific Functional Scale, Foot Function Index SPPS

10.7717/peerj.21280/supp-6 Supplemental Information 6 Data from all subjects recoded over time for outcome measurements quality of life and ultrasound SPSS

10.7717/peerj.21280/supp-7 Supplemental Information 7 Raw data from all the participants SPSS

## Additional Information and Declarations

### Data Availability

The following information was supplied regarding data availability: The data is available in the Supplemental Files.
