# Supplementary material for: Pain relief in plantar fasciitis within 6–8 weeks using (ortho)manual therapy of the foot joints: a prospective cohort study
Source: PeerJ. 2026 May 25;14:e21280. doi: 10.7717/peerj.21280 (PMC13218342; doi:10.7717/peerj.21280)
Supplement: Supplemental Information 1 [file peerj-14-21280-s001.docx]

# Appendix 1 (Ortho)Manual Therapy Techniques

If necessary, start with traction of the toes. This provides possible relaxation in the foot muscles and makes other manipulations easier.

## **Fibula Proximal**

### *Manual Technique 1*

Patient's starting position: Supine. For this technique, the patient must be able to fully flex the knee.

Therapist's starting position: On the side to be treated.

Bend the knee and hip. The hip should be flexed beyond 90°. Ask the patient if he/she wants to let the leg “fall” outwards in a relaxed manner. Let the leg rest against you.

Place your front hand in the hollow of the knee, with the thumb resting against the fibula head. The hand and fingers point at an angle of approximately 45°. Grasp the lower leg with your back hand and place the foot and lower leg in external rotation. Now make a short, quick movement with the lower leg towards maximum flexion of the knee.

### *Manual Technique 2*

Patient's starting position: Supine. The side to be treated stands with the knee raised and the foot flat on the couch.

Therapist's starting position: On the side not to be treated.

Place the middle fingers of both hands around the proximal fibula head like a hook and give a firm “pinch” to translate the fibula head forward.

Please note n. peroneus communis.

### *Hammer & Thrift*

Depending on the position of the fibula, an up/down/back/forward translation can be performed.

## **Talocrural Joint**

*Manual Technique 1*

Patient's starting position: Supine

Therapist's starting position: At the foot end in a sitting or standing position. Place the bench at about elbow height.

Place the foot to be treated in 90° dorsiflexion. Place both hands around the foot, with the little fingers at the level of the talus. Hold the foot securely. Use the thumbs on the bottom to keep the foot in a good dorsiflexed position. Now make a hard fast traction of the foot. Move the elbows backwards in a horizontal position (rowing motion), so that you do not pull the foot up or down.

Explain to the patient that traction can be felt in the knee, hip or low back. Pulling the foot automatically means pulling the entire leg. It is often not painful. For people with a lot of back pain, it sometimes helps to pull in the navel or to put the foot of the other leg on the bench (knee flexion).

## **1st Ray**

*Manual Technique*

Patient's starting position: Supine

Therapist's starting position: At the foot end

Place the thumbs behind the metatarsal head II. Hold the foot with the rest of the fingers, with the middle finger resting on top of the navicular bone. Keep the foot in dorsiflexion and internal rotation. Now make a quick traction movement towards yourself and up. You push against the CM II. You pull the medial cuneiform bone off the navicular bone.

To manipulate metatarsal 1 versus medial cuneiform, grasp the cuneiform in the snout grip with your thumb and index finger. With the other hand, grab the big toe and make a quick up and down movement.

### *Hammer & Thrift*

With the thrift you approach the foot from the bottom. Place the thrift on the inner edge of the cuneiform and strike the cuneiform off the navicular.

For the cuneiform to the metatarsal, approach the foot from the top. Place the thrift on the lateral edge of metatarsal 1 just above the joint space and strike off the metatarsal.

## **2nd/3rd/4th ray relative to tarsus**

## *Manual Technique*

## Patient's starting position: Supine

# Therapist's starting position: At the foot end

# Move the thumbs behind the head of the beam you want to loosen. Hold the foot with the rest of the fingers, with the middle fingers lying on top of the cuneiform bone and cuboid bone. Keep the foot in dorsiflexion and internal rotation. Now make a quick traction movement towards yourself. You always push against the metatarsal caput of the ray in question. You pull the metatarsals off the tarsus.

### *Hammer & Thrift*

### With the thrift you can approach the foot from above or below. Place the thrift on the medial edge of the radius of interest and strike away from the tarsus.

## **5th ray/cuboid**

*Manual Technique*

Patient's starting position: Supine

Therapist's starting position: At the foot end

Place the thumbs behind the metatarsal head. Grasp the foot with the rest of the fingers, with the middle finger on top of the cuboid bone. Keep the foot in dorsiflexion and external rotation. Now make a quick traction movement towards yourself and yourself. You push against the CM V. You subtract the metatarsal V from the cuboid bone.

To manipulate the cuboid, place the ankle in dorsiflexion, but the foot in plantarflexion. This relaxes the band structure under the cuboid.

### *Hammer & Thrift*

With the thrift you approach the foot from the bottom. Place the thrift on the inner edge of the 4th and/or 5th ray and strike it away from the cuboid bone.

For the cuboid bone it depends on the rotation that the bone piece has made. With the thrift you make the opposite rotation to put the joint back in the right place. You do this from the bottom.

## **Cuneiforme**

*Manual Technique*

Patient's starting position: Lying prone, knee bent

Therapist's starting position: On the side to be treated

Hold the heel bone. With this same hand you palpate the blockages of the cuneiforme. These feel like pebbles. The muscle mass is also often somewhat thicker during a blockage. In the event of a blockage, push the cuneiform upward with your finger/thumb, while using the other hand to tension the arch of the foot slightly towards dorsiflexion and then make a quick movement towards plantar flexion.

### *Hammer & Thrift*

Use the thrift to approach the foot at the bottom. Place the punch on the relevant cuneiform and drive the “wedge” upwards.

## **Subtalar Joint**

*Manual Technique*

Patient's starting position: prone, feet over the edge

Therapist's starting position: At the foot end

Place the foot in plantar flexion and inversion. Grasp the calcaneus with one hand and the foot with the other. Now make a short and quick traction movement.

### *Hammer & Thrift*

Use the thrift to approach the foot at the rear. Tap the calcaneus medially, dorsally and laterally. Do this on the upper edge and lower edge of the calcaneus.
