# Supplementary material for: Pain relief in plantar fasciitis within 6–8 weeks using (ortho)manual therapy of the foot joints: a prospective cohort study
Source: PeerJ. 2026 May 25;14:e21280. doi: 10.7717/peerj.21280 (PMC13218342; doi:10.7717/peerj.21280)

Appendix 2 Procedure Corrective Insoles

**Scanning procedure**

Used Software System: Paracontour

Feet are scanned while sitting

Scans are made from the best foot

Standard use of 50 shore hardness of the highest EVA (Ethyleen Vinyl Acetaat) quality

Standard use of bottom material realux

Standard use of leather sole cover

**Modelling procedure**

*Step 1- Smoothing the sole and shaping the Medial Internal Comfort (MIC)*

Action MIC: Correction calcaneus towards eversion

Limit settings

Distal: Caput Metatarsal (CM) 1

Proximal: +/- 1cm proximal to the medial malleolus

Medial: Forms a straight line between the distal and proximal limits

Lateral: An asymmetrical convex curved line with the widest point approximately to the axis of the sole

Highest point: navicular bone descending to CM 1

The MIC is automatically determined in terms of height by the system

Specific characteristic of the correction sole of VoetPortaal: Plantar touching parts from the hindfoot to the toes are smoothed at “0”. The arc remains stationary, creating the MIC. The height of the MIC is automatically determined by the system. From the navicular bone, the bulge is removed in a straight line to “0” so that it cannot touch the medial arch of the foot. Please note that the tendon of the peroneus longus muscle must not be touched anywhere. This is done in step 4.


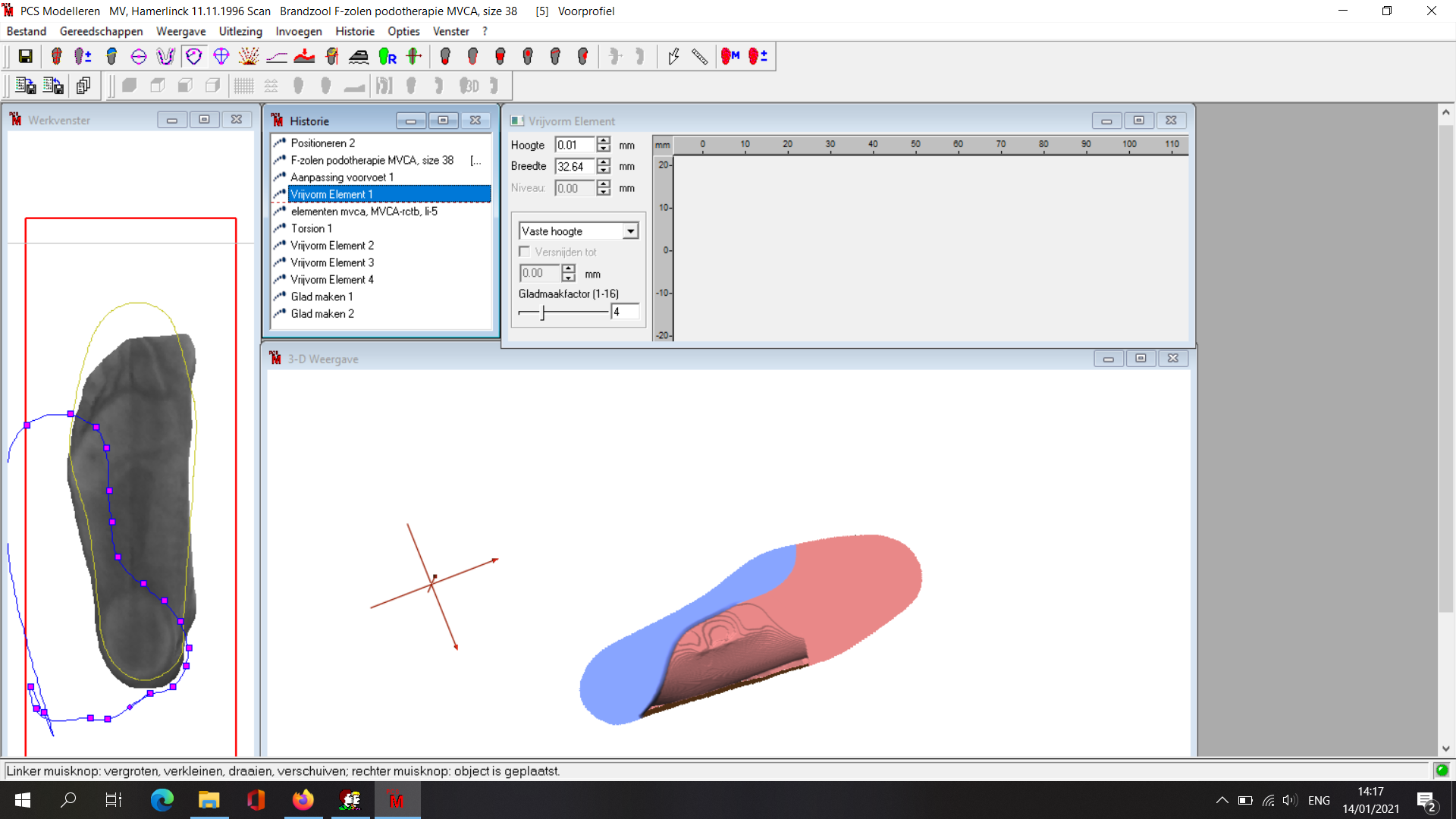


*Step 2 – Placing the Pronator Anterior (PA)*

Action PA: Stabilization on the 4-5 rays, which improves the function of the peroneal muscle on the 1st ray.

Limit settings

Distal: Retrocapital CM 1 to CM 5

Proximal: Line of Lisfranc

Medial: Oblique line between the distal and proximal limits

Specific characteristic of the correction sole of VoetPortaal is that the PA is formed from an RCTB and that part is then smoothed again. This is done in step 4.


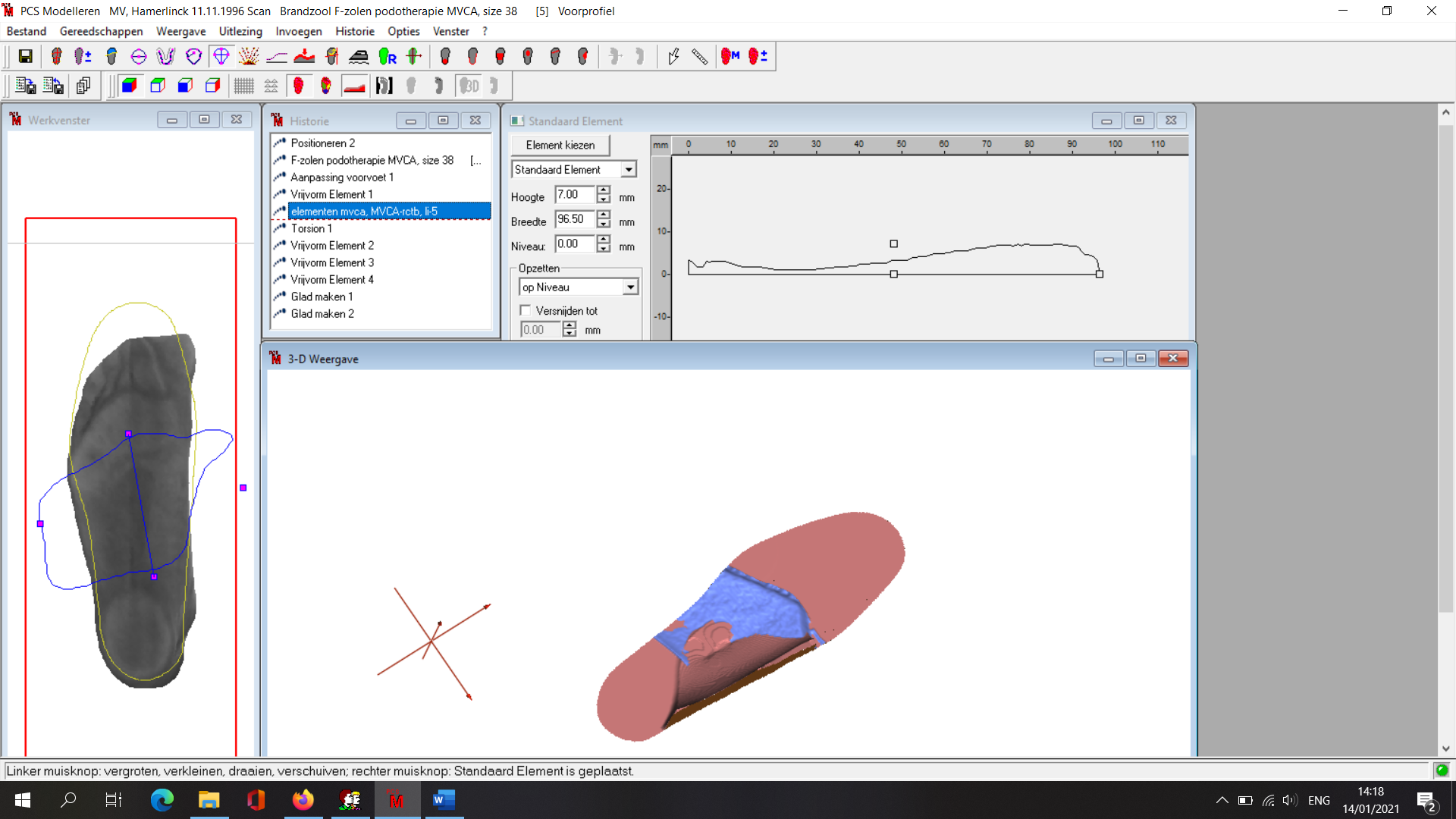


*Step 3 – Placing the supination posterior stimulator (SPSi)*

Action SPSi: Stimulation of supination in the Subtalar Talar Joint (STJ), inhibition of pronation in the STJ.

Limit settings

From the highest point descending to Metatarsale 1.


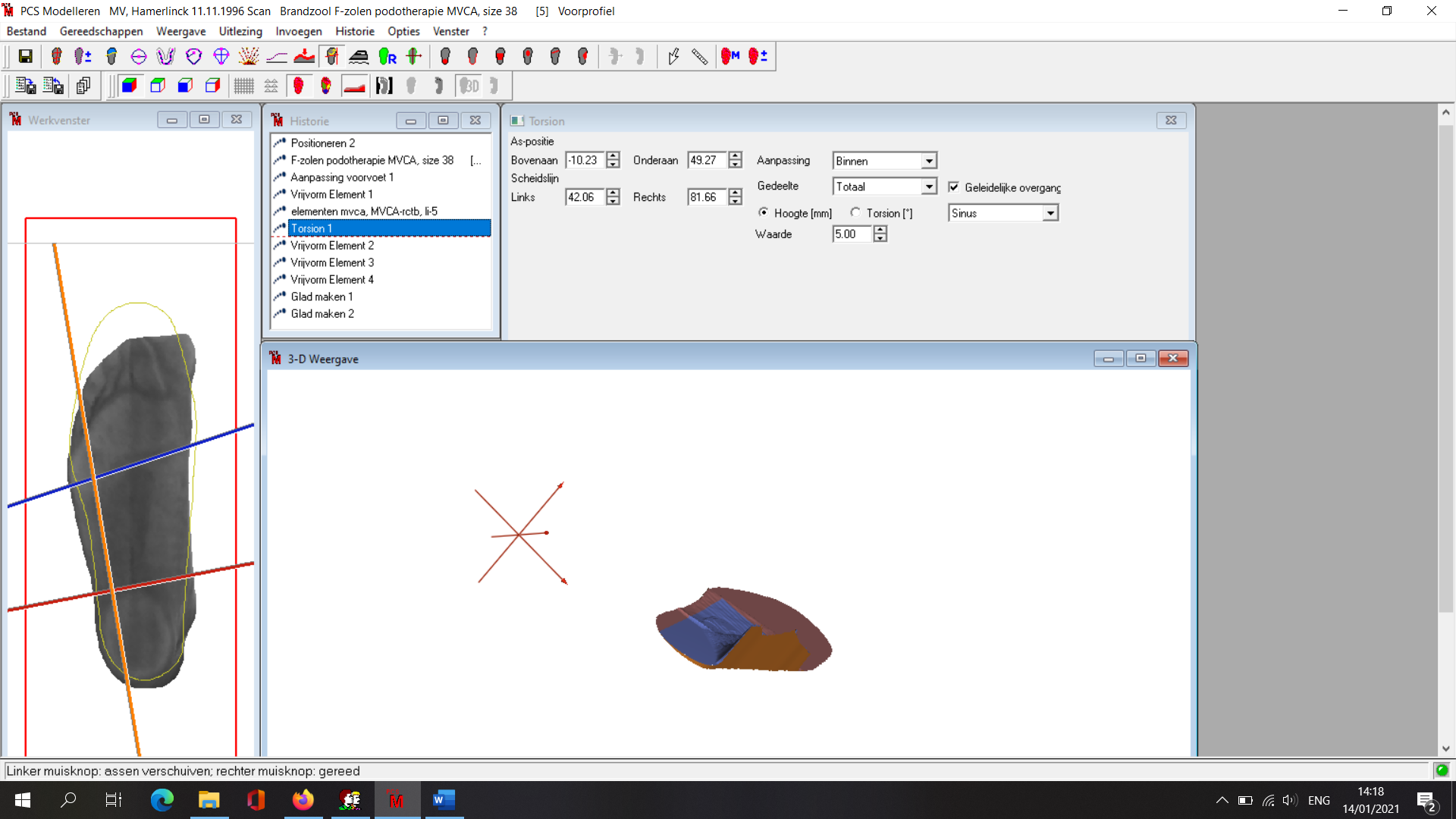


*Step 4 - Smoothing parts of the sole that hinder the functioning of the peroneal muscle.*


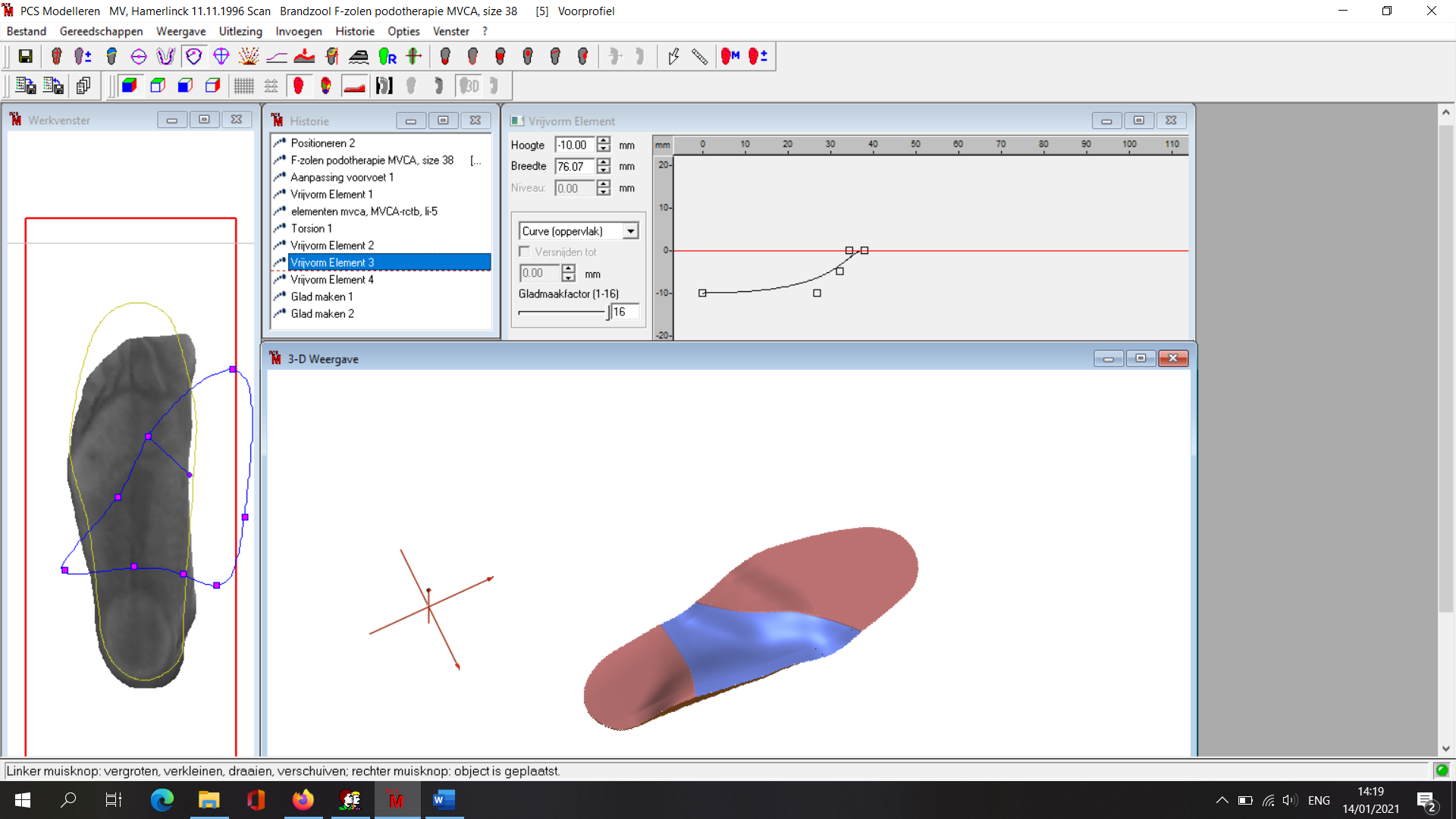


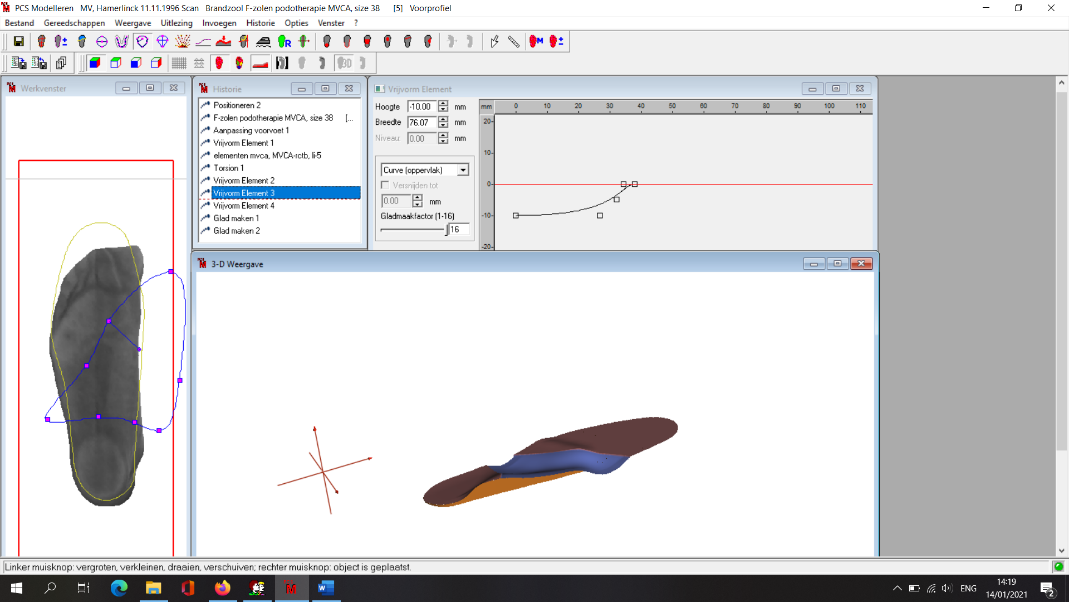

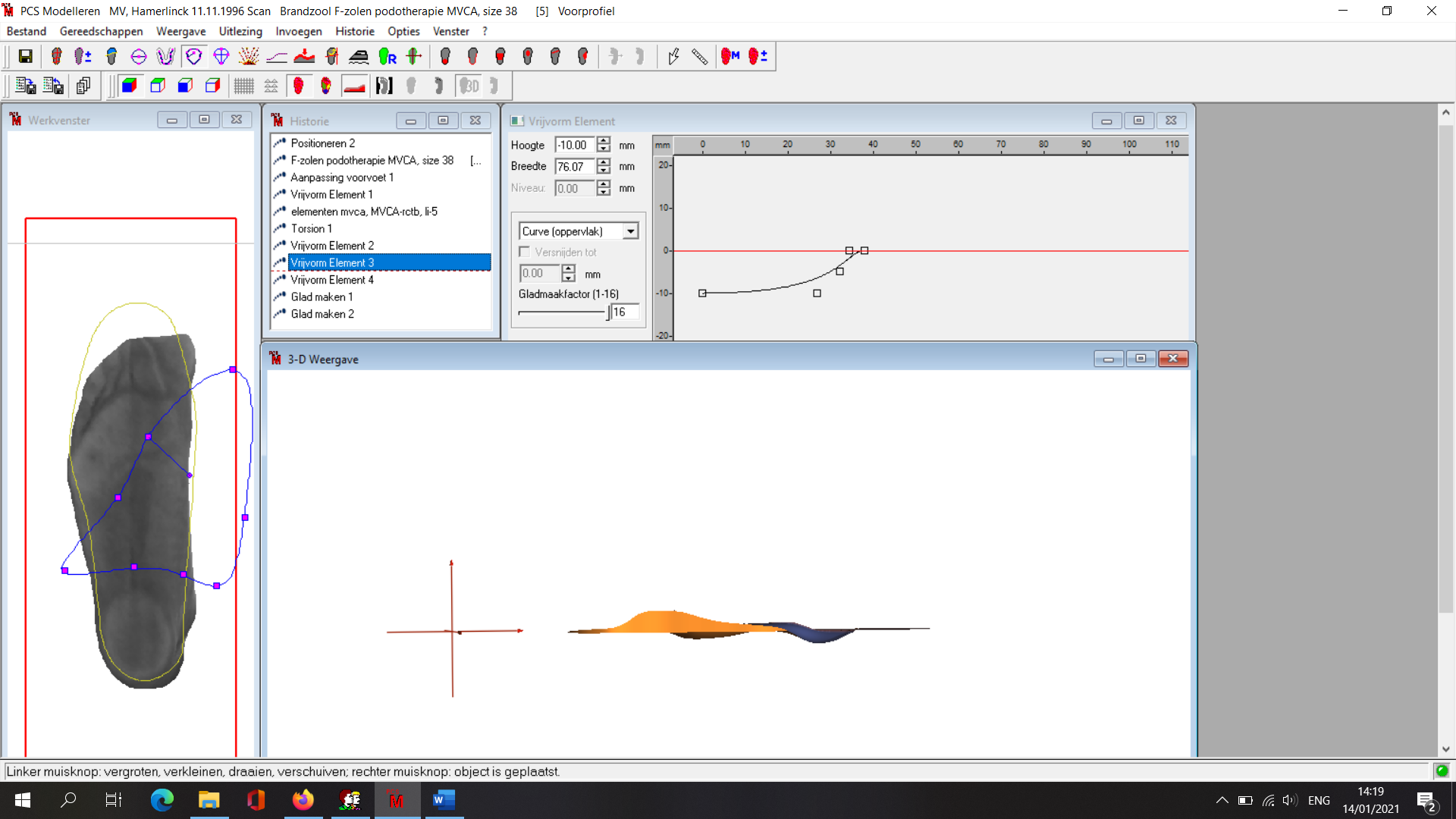


*Step 5 – The end result*


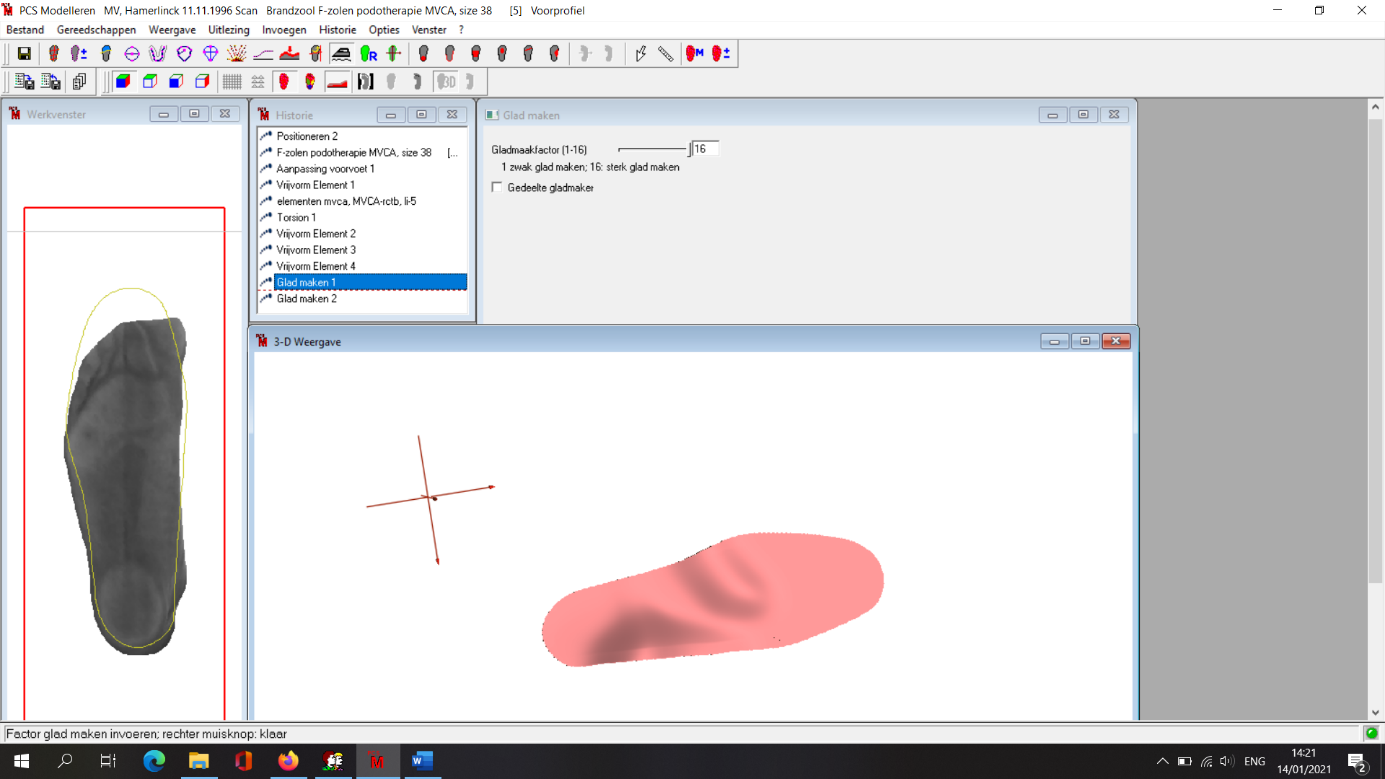

Supplement: Supplemental Information 2 [file peerj-14-21280-s002.docx]
