# Supplementary material for: Pain relief in plantar fasciitis within 6–8 weeks using (ortho)manual therapy of the foot joints: a prospective cohort study
Source: PeerJ. 2026 May 25;14:e21280. doi: 10.7717/peerj.21280 (PMC13218342; doi:10.7717/peerj.21280)
Supplement: Supplemental Information 3 — English-language codebook [file peerj-14-21280-s003.docx]

| **Codebook** | | |
| --- | --- | --- |
| **Column Name** | **Description** | **Possible Values / Units** |
| PatienID | Unique ID number of participant | Numeric |
| Uitvaller | Drop Out | “1” = No, “2” = Yes  Drop outs in this data file were the participants who did not complete the study. |
| Leeftijd | Age of participant | Numeric (years) |
| Geslacht | Gender of participant | "1" = Male, "2" = Female |
| Opleiding | Education of participant | “1”= High School, “2”= Vocational training (MBO level), “3”= University of Applied Sciences (Bachelor level), “4”= Research University (Bachelor/master level), “5”= Postdoctoral level |
| Lengte | Height of participant | Numeric (cm) |
| Gewicht | Weight of participant | Numeric (kg) |
| BMI | Body Mass Index of participant | Numeric |
| Beroep | Occupation | “1”= standing, “2”= sitting, “3”= standing/sitting, “4”= None/Pension |
| Comorbiditeiten | Comorbidities | Number of mentioned comorbidities by participant for example diabetes mellitus II. |
| Afspraken | Appointments | Number of appointments in total. |
| Afspraak T1/T3/T5/T6 | Appointment T1/T3/T5/T6 | Date of participant’s appointments for the different measurement moments. |
| Klacht_zijde | Affected side | “1”= Left, “2”= Right, “3”= both |
| Klacht_lok_re  Klacht_lok_li | Most painful location on the foot at the right and or left foot | “1”= Fascia Plantaris region, “2”= Directly underneath calcaneus, “3”= Tuberositas medialis of the calcaneus, “4”= Outside border of the heel, “5”= None.  When the value is for example 24; this means location 2 and 4. |
| Klacht_duur | Duration of the complaints | “1”= Since couple of days, “2”= Since couple of weeks, “3”= Since couple of months, “4”= 6 months, “5”= > 6 months, “6”= > 1 year |
| Specialisten | Specialists the participant has consulted before | “1”= GP, “2”= Physiotherapist, “3”= Podiatrist, “4”= Orthopaedic Surgeon, “5”= None/other  When the value is for example 24; this means specialist 2 and 4. |
| Therapieën | Treatments the participant received before | “1”= Pain medication, “2”= Exercises, “3”= Shockwave, “4” = Dry needling, “5”= Massage, “6”= Manual therapy, “7”= Corticosteroid injection, “8”= Taping, “9”= Custom insoles, “10”= Confection insoles, “11”= Shoe advice, “12”= Heel pad, “13”= Strassburg sock, “14” = None/other  When the value is for example 24; this means therapy 2 and 4. When the value is -14; this means therapy 1 and 4, not 14. |
| Klachten_nu Klachten_verleden | Self-reported musculoskeletal complaints at the moment of the first visit or in the past | “1”= Ankle, “2”= Knee, “3”= Groin, “4”= Hip, “5”= Lower Back, “6” = Thoracic Spine, “7”= Cervical Spine, “8”= Shoulder, “9”= None  When the value is for example 24; this means complaint 2 and 4. |
| T1_Echografie_re  T1_Echografie_li  Also for T5 and T6 | T1 Ultrasound right foot  T1 Ultrasound left foot | Result of the ultrasound in mm at the specific measurement moment. |
| DF_rechts_beperkt  DF_links_beperkt | Less than 10 degrees of dorsiflexion of the ankle joint for the left or right foot | “1”= Yes, “2”= No |
| Formule | Candidate for specific spine treatments | “1”= Yes, “2”= No |
| T1_SF12_PCS  Also for T5 and T6 | Physical Component Summary score from the SF-12 health questionnaire | A lower PCS score indicates poorer physical health and more limitations in daily life. The normative group is the American population from 1995. A score of 50 with a standard deviation of 10 is considered average. |
| T1_SF12_PCS_USA  Also for T5 and T6 | Deviation from the American population mean (50) on SF-12 PCS | The mentioned number is the deviation above or under 50. |
| T1_SF12_MCS  Also for T5 and T6 | Mental Component Summary score from the SF-12 health questionnaire | A lower MCS score indicates poorer mental health and more problems in this area. The normative group is the American population from 1995. A score of 50 with a standard deviation of 10 is considered average. |
| T1_SF12_MCS_USA  Also for T5 and T6 | Deviation from U.S. population mean (50) on SF-12 MCS | The mentioned number is the deviation above or under 50. |
| T1_Squash  Also for T5 and T6 | Short QUestionnaire to ASsess Health-enhancing physical activity | Variables based on the SQUASH questionnaire (Short Questionnaire to Assess Health-enhancing Physical Activity) were collected but not included in the final analyses due to lack of relevance to the outcome. |
| T1_FFI_Pain  T1_FFI_Dis  T1_FFI_Act  T1_FFI_Tot  Also for T3, T5 and T6 | Foot Function Index  - Pain subscale score  - Disability subscale score  - Activity Limitation subscale score  - Total Score | Scores range from 0 to 100, with higher scores indicating more severe symptoms or limitations. |
| DN4 | Douleur Neuropathique en 4 questions | Used as a screening tool to identify neuropathic pain. The total score ranges from 0 to 10. A score of **≥4** is considered indicative of neuropathic pain. |
| T1_EQ5D  Also for T5 and T6 | Measures health-related quality of life. It assesses five dimensions of health: mobility, self care, usual activities, pain/discomfort, anxiety/depression | The EQ-5D-3L is used: 3 levels per dimension (no problems, some problems, extreme problems).  When the value is for example 21123: this means some problems on the first dimension, no problems on the second dimension etc. |
| T1_EQ5D_VAS | The EQ-5D includes a Visual Analogue Scale (VAS) | Participants rate their overall health from 0 (worst imaginable health) to 100 (best imaginable health). |
| T1_EQ5D_index | EQ-5D utility index score | Calculated from dimension responses using a country-specific value set; 1.0 = full health (best possible score), 0 = worst possible score. |
| T1_NPRS_Rust  T1_NPRS_Act  T1_NPRS_Stap1  Also for T3, T5 and T6 | Numeric Pain Rating Scale  - In rest  - During activities  - With the first step in the morning | Scores range from 0 to 100, with higher scores indicating more pain. |
| T1_PSK_Act1  T1_PSK_Act2  T1_PSK_Act3  T1_PSK_Total  Also for T3, T5 and T6 | The Patient Specific Functional Scale, activity 1,2,3 and total. Not every participant mentioned three activities. | Scores range from 0 to 10 per activity, with higher scores indicating more difficulty performing the activity. For th total PSFS score the item scores of the activities are added up and divided through the number of mentioned activities. |
| T5_SRLS  Also for T6 | Self-Reported Likert Scale where the participants rated their own perception on improvement. | “1”= Worse than before, ”2”= No recovery, “3”= Little improvement, “4”= Moderate improvement, “5”= Significant improvement, “6”= Strongly recovered, “7”= Fully recovered |
